# Patterns of Mortality for Children and Adolescents in Early 20th‐Century Alaska

**DOI:** 10.1002/ajhb.70347

**Published:** 2026-10-11

**Authors:** Taylor P. van Doren, Delaney Glass, Christiane Scheffler, Michael Hermanussen, Lisa Sattenspiel

**Affiliations:** ^1^ Department of Sociology & Anthropology High Point University High Point North Carolina USA; ^2^ Department of Anthropology University of Toronto Toronto Ontario Canada; ^3^ Institute of Biochemistry and Biology, Human Biology, University of Potsdam Potsdam Germany; ^4^ Altenhof Germany; ^5^ Department of Anthropology University of Missouri Columbia Missouri USA

**Keywords:** age‐based mortality, child and adolescent health, early 20th century, multinomial logistic regression, proportionate mortality

## Abstract

**Objectives:**

Odds of death from specific causes in infants, children, and adolescents in Alaska have not been quantified with vital records. We analyze the odds of death from 12 causes in individuals ≤ 18 years.

**Methods:**

We fit a multinomial logistic regression model (*n* = 12 904 death records) controlling for Indigeneity, age, sex, and year of death to predict the odds of specific causes of death.

**Results:**

Alaska Native individuals were most likely to die from tuberculosis and respiratory infections, while non‐Alaska Native individuals were most likely to die from early infant health problems and accidents/violence. Infectious diseases and nutritional and endocrine disorders were most likely in ages 0–5, while accidents/violence, cardiovascular issues, and tuberculosis were most likely in ages 10–18.

**Conclusions:**

Proportionate mortality and odds of death differed significantly, reflecting the different structures of Alaska Native versus non‐Alaska Native populations in the 20th century and reinforcing demographic observations in the historical record.

## 1 Background

Mortality is age‐ and sex‐specific across the lifespan. Age‐, sex‐, and cause‐specific mortality rates in individuals ≤ 18 years are well studied in modern and historical contexts with the integration of vital and census records. For example, infectious diseases are particularly dangerous for infants and children, especially those of the respiratory and gastrointestinal systems (Birn et al. 2010; McGregor et al. 1961; Patterson 1979). However, the statistical odds (probability) of death from specific causes in history within populations with individual death records are relatively understudied. Epidemiological transition research shows that improvements in population health were linked to substantive improvements in maternal and child health (Omran 1971), though these transitions were dependent on diverse socioeconomic and sociopolitical factors that were not uniform everywhere (Santosa et al. 2014).

Alaska's 20th century health profile should not be expected to match the transitions observed in industrialized nations, as most of the population was living in thousands of remote localities across the temperate to Arctic ecological spectrum (Allhoff and Goleman 2020). Generalizations about population health improvements, which were theorized with national‐level population data, cannot be easily applied to populations that would benefit from a lower level of demographic analysis, especially in Arctic populations (van Doren et al. 2023). The scope of mortality risks experienced in childhood and adolescence was described in the historical record (Fortuine 1989), where those related to tuberculosis in Alaska Native (AN) Peoples are especially noteworthy (35.5% of all causes for all ages were attributed to tuberculosis between 1926 and 1930 in Alaska Native Peoples, while only 4.7% of all deaths were related to tuberculosis in settlers) (Fellows 1934).

Alaska is an Arctic and sub‐Arctic region with a diverse population of AN peoples. During the early 20th century, half the population was AN, and the other half comprised colonial settlers and industry laborers (Roderick 2010; Williams 2001, 2009). At the time, the non‐AN population was predominantly adults with comparably few children and adolescents, while the AN population was represented by individuals of all ages. These differences in demographic structure likely led to distinct patterns of mortality in individuals ≤ 18 years, as contemporary age‐based mortality rates show that most mortality in AN populations across Alaska occurred in individuals < 20 years (Fellows 1934).

Here, we identify general patterns of probability of death based on Indigeneity, age, and sex during childhood and adolescence in Alaska for the first time with vital records. We hypothesize that the non‐AN population will have a high probability of infectious causes in infancy and a lower probability of infectious causes in older adolescence, whereas AN children and adolescents will be most affected by infectious diseases, especially tuberculosis. The ultimate goal of this research is to identify these patterns for the development of future in‐depth demographic and epidemiological research on cause‐specific mortality in Alaskan children and adolescents.

## 2 Materials and Methods

We used individual death records of people aged ≤ 18 years who died from any recorded cause from 1910–1939 (*n* = 12 904). PDFs of original death records were obtained from the Alaska Bureau of Vital Statistics, where each record included two pages of demographic, biometric, and family data. This dataset was transcribed by hand from 2021 to 2024 by authors T.P.v.D. and L.S. The demographic makeup of the sample is 41.5% AN females, 45.5% AN males, 5.5% non‐AN females, and 7.5% non‐AN males, indicating a clear bias toward AN children and adolescents in the death records. The demographic makeup of non‐AN individuals in this sample is overwhelmingly comprised of white settlers from Canada, the United States, and Europe; there are fewer than 10 non‐AN individuals whose recorded race is either Black or Asian.

There were 3956 unique causes of death, most of them suggesting plausible diagnoses, while many listed symptoms. In lieu of using International Classification of Disease categories, which are not recommended for use in historical data by historical demographers, historical epidemiologists, and medical historians (Alter and Carmichael 1996), we prioritize a categorization system appropriate for the historical context that broadly categorizes deaths into (a) organ systems, (b) accidents/violence, and (c) infectious diseases (Janssens and Devos 2022). Due to the age categories of interest in this paper, we also differentiate two additional categories for analysis: diseases specific to childhood and early infant health problems. The result is 12 categories which—aside from surgical interventions—each comprise at least several hundred cases. The final 12 categories and their frequencies are summarized in Table 1. Further detail on cause of death categories is in Supplementary Table 1.

**TABLE 1:** Summary of the 12 major causes of death categories constructed from the individual death records (Alaska death register, 1910–1939, *n* = 12 904).

Cause of death category	Frequency (% of Total)		Significance	
	Alaska Native	Non‐Alaska Native	*z*‐score	*p*‐value^a^
Tuberculosis	3037 (27.0)	129 (7.1)	17.2	< 0.001***
Unknown/ill‐defined	1301 (11.6)	87 (5.2)	7.9	< 0.001***
Respiratory system	2676 (23.8)	224 (13.4)	9.6	< 0.001***
Childhood diseases	855 (7.6)	64 (3.8)	5.6	< 0.001***
Digestive system	527 (4.7)	67 (4.0)	1.3	0.23
Nutritional and endocrine disorders	198 (1.8)	36 (2.2)	−1.1	0.27
Other infectious diseases	464 (4.1)	81 (4.8)	−1.3	0.18
Cardiovascular diseases	129 (1.2)	36 (2.2)	−3.4	< 0.001***
Less frequent causes	399 (3.6)	94 (5.6)	−4.1	< 0.001***
Surgical intervention	45 (0.4)	21 (1.3)	−4.6	< 0.001***
Accidents/violence	662 (5.9)	307 (18.3)	−18.0	< 0.001***
Early infant health problems	937 (8.3)	528 (31.5)	−27.9	< 0.001***
Total	11 230	1674		

*Note:* The frequency of each cause of death and percent contribution of each category are stratified by ethnicity group (AN vs. non‐AN). Calculated two‐proportion *z‐*scores between the proportion of cause of death to overall deaths between AN and non‐AN populations, where a positive *z*‐score indicates higher AN proportionate mortality and a negative *z*‐score indicates higher non‐AN proportionate mortality. Associated corrected *p* values are also reported. The table is sorted in descending order by *z*‐score. Significance codes: *p* < 0.001 = “***”.

^a^ *p* values reported are adjusted using the Benjamini–Hochberg correction.

We estimate significant differences in proportionate mortality for causes of death between AN and non‐AN populations using two‐proportion *z*‐scores, which measure statistically identifiable differences between two independent proportions. We include a *p* value correction to protect against a high false discovery rate using the Benjamini–Hochberg method. Finally, we assess the probability of death for each of the 12 major causes. A multinomial logistic regression model was fit according to Equation (1):

(1) $$\begin{array}{c} \log\left(\frac{p\left(j\right)}{p\left(J\right)}\right)=\beta_{0}+\beta_{1}x_{\text{ethnicity group}}+\beta_{2}x_{\mathrm{sex}}\\ \;+\beta_{3}x_{\mathrm{age}\;\mathrm{at}\;\text{death}}+\beta_{4}x_{\text{year of death}}+\varepsilon \end{array}$$

where the expression $\left(\frac{p\left(j\right)}{p\left(J\right)}\right)$ is the probability of a cause of death against the baseline cause; age at death and year of death are continuous variables; ε is the model error; and the categorical variable is:

$$x_{\text{ethnicity}}=\left\{\begin{array}{c} 0:\text{Alaska Native}\\ 1:\text{non‐Alaska Native} \end{array}\right.$$

## 3 Results

Table 1 reports the results of proportionate mortality between AN and non‐AN groups. Proportionate mortality is similar for only three causes: illnesses of the digestive system, nutritional and endocrine disorders, and other infectious diseases (all *p* > 0.05). For AN individuals, the two largest contributions to mortality are tuberculosis (27.0%) and respiratory diseases (23.8%). For non‐AN individuals, the two largest contributions are early infant health problems (31.5%) and accidents/violence (18.3%).

Figure 1 illustrates age‐ and sex‐dependent odds of mortality predicted with the multinomial regression model. AN individuals were more likely to die from tuberculosis, respiratory system diseases, unknown/ill‐defined diseases, childhood diseases, and digestive system diseases, while non‐AN individuals are more likely to die from nutritional and endocrine disorders, surgical interventions, other infectious diseases, cardiovascular diseases, less frequent causes, accidents/violence, and early infant health problems. Figure 1A shows the difference in probability of a specific cause between AN and non‐AN groups stratified by sex. For example, an AN female is 18.0% more likely to die of tuberculosis compared to a non‐AN female (*p* < 0.001). On the other hand, a non‐AN female is 17.5% more likely to die of early infant health problems than an AN female (*p* < 0.05). For males and females, differences in probability of mortality for nutritional and endocrine disorders and digestive system diseases are small (< 0.5% difference), though the model predicts statistically significant differences in odds of mortality.

**FIGURE 1:**
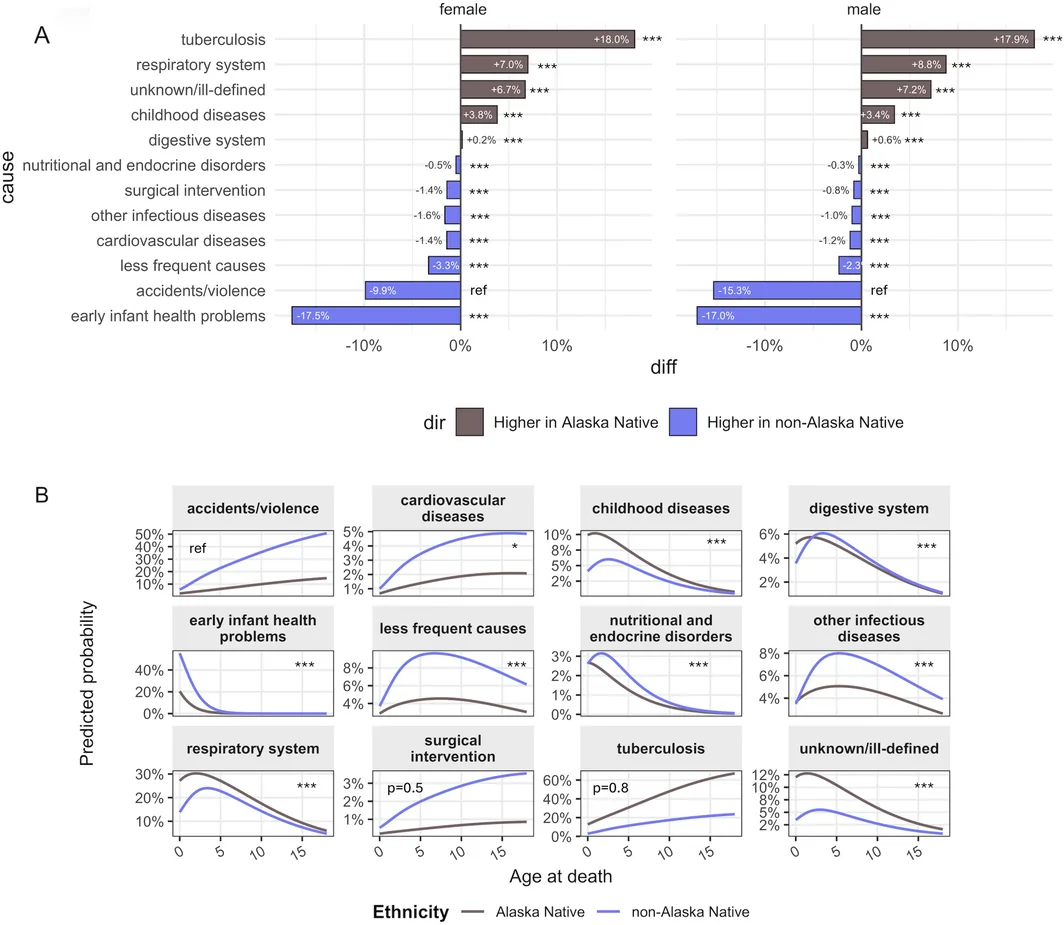
Illustrated results of the multinomial logistic regression model to predict the probability of death from one of 12 major cause of death categories in early 20th century Alaska. (A) The difference in predicted odds in AN and non‐AN individuals for each cause of death faceted by sex. (B) The probability of death for each age, controlling for ethnicity group. Significance values estimated by the regression model are included in each facet, where *** = *p* < 0.001; ** = *p* < 0.01; * = *p* < 0.05, and (.) = *p* < 0.10.

In Figure 1B, probability of death in ages 0–5 peaks in common childhood causes, digestive system diseases, early infant health problems, nutritional and endocrine disorders, respiratory diseases, and unknown/ill‐defined causes, regardless of ethnicity. Probability of death in ages 10–18 peaks for accidents/violence, cardiovascular diseases, surgical interventions, and tuberculosis. Age strongly predicts probability of death from any cause (all *p* < 0.001, except cardiovascular disease, *p* = 0.034), apart from surgical interventions and tuberculosis (*p* = 0.5 and 0.8, respectively). Death year is also a significant predictor of cause of death with small per‐unit changes in OR, where the odds attributed to a specific cause of death decrease as the year of death increases, indicating that there is a significant (though minimal) shift in the odds of mortality over time (ORs = 0.95–1.00, all *p* < 0.001).

## 4 Discussion

The results of this study show that AN children and adolescents were significantly more likely to die from primarily infectious disease causes of death, while non‐AN individuals suffered from accidents/violence. Additionally, the odds of death from infectious diseases, digestive system diseases, and nutritional and endocrine disorders declined precipitously after age 5. This pattern adheres to the general trend observed in modern (i.e., today's) age‐specific patterns of death, where highest risk is in infants. However, differences in odds of death in infancy between AN and non‐AN individuals may not represent true differences, but rather evidence of bias in how infant mortality is/was recorded. Systematic under‐reporting of Native infants leads to the underestimation of non‐white causes of infant mortality (Farley et al. 1995), which would overestimate the gap in mortality between AN and non‐AN individuals. The significantly higher proportion of early infant health problems in non‐AN individuals may indicate this phenomenon in Alaska. Beyond systematic data collection barriers, we must also acknowledge that cultural adaptation and resilience in infant and childhood health could be strong social determinants of health for AN families that protect against increased risk of death in the earliest age classes (James et al. 2021). However, there is very little research on this topic, and what research does exist amalgamates all American Indian and Alaska Native individuals in the United States with late 20th and early 21st century data (e.g., Oré et al. 2016), making it difficult to tease apart relevant cultural contexts for historical Alaska Native Peoples.

The high proportions and odds of death for AN children and adolescents from tuberculosis and respiratory diseases align with the historical record. Tuberculosis was a leading cause of overall mortality since at least the 19th century, especially for the AN population (Comstock and Philip 1961; Fortuine 1989, 2005). Here, odds of death from tuberculosis were significantly higher for AN individuals, and both male and female AN individuals have ~18% higher odds of tuberculosis death than non‐AN individuals (Figure 1a). Additionally, 1910–1939 captures the 1918 flu (1918–1920), during which AN communities were among the most severely affected regions of the world (Mamelund et al. 2013; Phillips‐Chan 2024). However, children aged 5–14 are at lower risk of death from influenza and pneumonia while those aged 0–2 are at the highest (Bhutta and Saeed 2008); these results reflect known age‐based patterns of influenza and pneumonia epidemiology.

Although there were ~50:50 AN to non‐AN individuals alive in Alaska during the early 20th century, proportionate mortality between these groups differed significantly (Table 1). For example, 50.4% of AN deaths were due to respiratory diseases and tuberculosis, while 32% of recorded non‐AN deaths were from infant health problems. Contemporary statistical censuses show there was a difference in population structure between AN and non‐AN individuals, primarily that most non‐AN were not, in fact, children (Institute for Social and Economic Research 1984). Historical demographic structure data do exist to expand upon this research to investigate demographic and epidemiological patterns of the early 20th century beyond the present study. Specifically, historical Alaskan census data provide population sizes and structures that allow for the calculation of standardized mortality rates, thus allowing for the quantification of epidemiological burden of specific causes of death rather than just comparisons of odds between and/or among subpopulations. Additionally, historical Alaskan censuses are often detailed down to the locality level, making region‐based comparisons of mortality possible. This would be particularly illuminating, as the historical disease‐scape of Alaska may be further understood through a region‐based analysis that prioritizes how space and place influence population health (Raymer et al. 2018). These opportunities for advancement could help adjust for the raw population structure discrepancies between AN and non‐AN children and adolescents.

## 5 Conclusion

The probability of AN infants, children, and adolescents suffering an infectious disease cause of death, especially tuberculosis, other diseases of the respiratory system, and malnutrition was significantly higher than the probability of a non‐AN individual of the same age. Rather, non‐AN individuals ≤ 18 years were most likely to die of early infant health problems and surgical interventions. This is the first time the likelihood of cause of death has been quantified with individual death records in this context. Future research can utilize other historical data sources to provide more in‐depth quantitative demographic analyses of cause‐specific mortality that can be comparable to contemporaneous child and adolescent populations.

## Author Contributions

**Taylor P. van Doren:** conceptualization, data curation, formal analysis, funding acquisition, investigation, methodology, project administration, software, visualization, writing – original draft preparation, writing – review and editing. **Delaney Glass:** conceptualization, formal analysis, methodology, writing – original draft preparation, writing – review and editing. **Christiane Scheffler:** conceptualization, data curation, methodology, supervision, writing – original draft preparation, writing – review and editing. **Michael Hermanussen:** conceptualization, data curation, formal analysis, methodology, supervision, writing – original draft preparation, writing – review and editing. **Lisa Sattenspiel:** data curation, funding acquisition, resources, supervision, writing – review and editing.

## Funding

This study was supported by the National Science Foundation Office of Polar Programs Postdoctoral Research Fellowship (#2219216) (to T.P.v.D.) and the University of Missouri College of Arts & Sciences Heart of Mizzou Endowment (to L.S.).

## Disclosure

No generative AI technology was used in the preparation of this manuscript.

## Ethics Statement

This research does not use human subjects data and was therefore determined exempt by both the Alaska Area IRB and the University of Alaska IRB. Individual death records become available for public research after exactly 50 years from the date of death, and Alaskan records are available for research with a signed data usage agreement and per‐year remuneration.

## Conflicts of Interest

The authors declare no conflicts of interest.

## Supporting information


**Supplementary Table 1** Historical cause‐of‐death categorizations of primary causes of death from the individual death records. The 12 broad categories used in this paper are shown with finer distinctions, while the nearly 4000 primary diagnoses from the death records are shown to which category they are attributed.

## Data Availability

The de‐identified data that support this research are available upon reasonable request from the first author. Metadata related to the data used in this research are available in the National Science Foundation Arctic Data Center (https://doi.org/10.18739/A2T727J2T).
